# Genomic GC-Content Affects the Accuracy of 16S rRNA Gene Sequencing Based Microbial Profiling due to PCR Bias

**DOI:** 10.3389/fmicb.2017.01934

**Published:** 2017-10-05

**Authors:** Martin F. Laursen, Marlene D. Dalgaard, Martin I. Bahl

**Affiliations:** ^1^Division of Diet, Disease Prevention and Toxicology, National Food Institute, Technical University of Denmark, Kongens Lyngby, Denmark; ^2^Department of Biotechnology and Biomedicine, Technical University of Denmark, Kongens Lyngby, Denmark

**Keywords:** ion torrent PGM, 16S rRNA gene sequencing, reproducibility, accuracy, mock community, genomic GC content

## Abstract

Profiling of microbial community composition is frequently performed by partial 16S rRNA gene sequencing on benchtop platforms following PCR amplification of specific hypervariable regions within this gene. Accuracy and reproducibility of this strategy are two key parameters to consider, which may be influenced during all processes from sample collection and storage, through DNA extraction and PCR based library preparation to the final sequencing. In order to evaluate both the reproducibility and accuracy of 16S rRNA gene based microbial profiling using the Ion Torrent PGM platform, we prepared libraries and performed sequencing of a well-defined and validated 20-member bacterial DNA mock community on five separate occasions and compared results with the expected even distribution. In general the applied method had a median coefficient of variance of 11.8% (range 5.5–73.7%) for all 20 included strains in the mock community across five separate sequencing runs, with underrepresented strains generally showing the largest degree of variation. In terms of accuracy, mock community species belonging to Proteobacteria were underestimated, whereas those belonging to Firmicutes were mostly overestimated. This could be explained partly by premature read truncation, but to larger degree their genomic GC-content, which correlated negatively with the observed relative abundances, suggesting a PCR bias against GC-rich species during library preparation. Increasing the initial denaturation time during the PCR amplification from 30 to 120 s resulted in an increased average relative abundance of the three mock community members with the highest genomic GC%, but did not significantly change the overall evenness of the community distribution. Therefore, efforts should be made to optimize the PCR conditions prior to sequencing in order to maximize accuracy.

## Introduction

Advances in Next Generation Sequencing (NGS) technology have revolutionized biological sciences during the last couple of decades. Within microbiota studies, PCR-based library preparation of the 16S rRNA gene and subsequent sequencing is commonly used to ascertain the microbial diversity and composition within various microbial habitats (Kuczynski et al., 2012). However, in such studies batch-to-batch variation may introduce bias that could significantly affect the results, i.e., due to the distribution of samples into different DNA extraction batches, PCR runs and sequencing runs (Leek et al., 2010). Another issue arises from the fact that the accuracy of the results depend on the choice of DNA extraction procedure, primer choice and PCR conditions, in addition to the sequencing technology itself (Pinto and Raskin, 2012; Tremblay et al., 2015; Walker et al., 2015; Fouhy et al., 2016). Here we present a simple strategy for library preparation based on non-degenerative universal PCR primers, a single PCR amplification (24 cycles) of the V3-region and subsequent sequencing on the Ion Torrent PGM platform using the Hi-Q chemistry. In order to evaluate this strategy for profiling microbial communities, we assessed the reproducibility and accuracy resulting from sequencing a well-defined and validated bacterial mock community, consisting of equimolar numbers of 16S rRNA genes contained in full-length bacterial genomes. We validated the performance based on overall numbers of OTUs identified following de-novo clustering at 97% homology in UPARSE (Edgar, 2013) and deduced community composition. Further, we evaluated the reproducibility across sequencing runs and accuracy in relative abundance estimates compared with the expected.

## Materials and methods

### Mock community and batch information

Genomic DNA from Microbial Mock Community B (Even, High Concentration), v5.1H, for Whole Genome Shotgun Sequencing, HM-276D was obtained through BEI Resources, NIAID, NIH as part of the Human Microbiome Project. The mock community consists of 200,000 16S rRNA genes embedded into the genomes of 20 bacterial species at equimolar concentration in terms of the 16S rRNA gene and was used as a template for separate library preparations and subsequent16S rRNA gene profiling on five different sequencing chips for evaluation of reproducibility and accuracy. A sixth library preparation and sequencing chip was included in order to improve accuracy by altering PCR conditions in the library preparation step.

### Primers and PCR amplification

The PCR amplification of the V3-region of the 16S rRNA gene was performed with 0.2 μl template DNA material (HM-276D), using 0.2 μl Phusion High-Fidelity DNA polymerase (Fisher Scientific, F-553L), 4 μl HF-buffer, 0.4 μl dNTP (10 mM of each base),1 μM forward primer (PBU 5′-A-adapter-TCAG-barcode-CCTACGGGAGGCAGCAG-3′) and 1 μM reverse primer (PBR 5′-trP1-adapter-ATTACCGCGGCTGCTGG-3′) in a 20 μl total reaction volume (primers were modified from Milani et al., 2013). Both the non-degenerative forward and reverse primers were identical to the corresponding region in all of the 20 different 16S rRNA genes represented in the multispecies mock community, ensuring that the primer choice did not contribute to PCR bias. Both primers (TAG Copenhagen A/S) were linked to sequencing adaptors and the forward primer additionally contained a unique 10 bp barcode (Ion Xpress™ Barcode Adapters) for each sample. The PCR program consisted of initial denaturation for 30 or 120 s at 98°C, followed by 24 cycles of 98°C for 15 s and 72°C for 30 s, and lastly 72°C for 5 min to allow final extension before cooling to 4°C. The PCR products were purified by use of HighPrep™ PCR Magnetic Beads (MAGBIO®, AC-60005) with a 96-well magnet stand (MAGBIO®, MyMag 96), according to the manufacturers recommendations. The DNA concentration of each PCR product was measured using the Qubit® dsDNA HS assay (Invitrogen™, Q32851).

### DNA sequencing and data handling

Sequencing of the 16S rRNA gene libraries was performed together with other biological samples on six separate occasions using the Ion OneTouch™ and Ion PGM systems with a 318-Chip v2 incorporating the Hi-Q chemistry in a 200 bp run with an average chip load 81.8% (range 76–86%), enrichment 100% and polyclonality 30.3% (range 17–40%). Sequencing data were deposited at the NCBI Sequence Read Archive with the Accession Number SRP110567 under BioProject PRJNA390244. The raw sequencing data were imported into CLC Genomic Workbench (version 8.5. CLC bio, Qiagen, Aarhus, DK) and reads were quality controlled, de-multiplexed according to barcode and trimmed to remove barcodes and 16S rRNA gene primers, maintaining only those reads for which both forward and reverse primers were identified with 100% identity (minimum alignment score 17/17, discard read when both primers were not found) and to discard reads below 125 bp or above 180 bp. Quality filtering (-fastq_filter, maxee 2.0), dereplication (-derep_fulllength), OTU clustering (-cluster_otus, minsize 6), mapping of reads to OTUs (-usearch_global, id 97%) and generation of the OTU table (python, uc2otutab.py) was performed within the UPARSE pipeline (Edgar, 2013). Taxonomy of the detected OTUs was assigned using the rdp classifier with confidence threshold 0.5 (recommended for sequences shorter than 250 bp) and the GreenGenes database v. 13.8 using the assign_taxonomy.py script incorporated in QIIME (Caporaso et al., 2010). Additionally, a BLAST search for all individual representative OTU sequences was performed against the 16S rRNA gene database at NCBI (Altschul et al., 1990). In order to investigate the effect of premature read truncation, we relaxed primer trimming (minimum alignment score 10/17), abolished all length and quality trimming as well as singleton removal. The resulting 5238 OTUs were classified as described above.

### Data analysis

The 31 detected OTUs were collapsed into the 20 mock community species based on the BLAST search against the 16S rRNA database at NCBI. Relative abundances were estimated by total sum scaling within each sequencing run. The reproducibility was assessed by the coefficient of variance (mean abundance/standard deviation). The accuracy was assessed by the log2 of the measured/expected relative abundance for each species in each sequencing run. The genomic GC content of the 20 species included in the mock community were obtained from the genome database from NCBI (https://www.ncbi.nlm.nih.gov/genome/). Evenness was calculated as Shannon index/log(20) using the diversity-function in the R package *vegan*. The correlation analysis (Spearman's Rank) and *t*-tests were performed with the GraphPad Prism software (v. 7.0, GraphPad Software Inc., La Jolla, CA).

## Results

**Figure 1:**
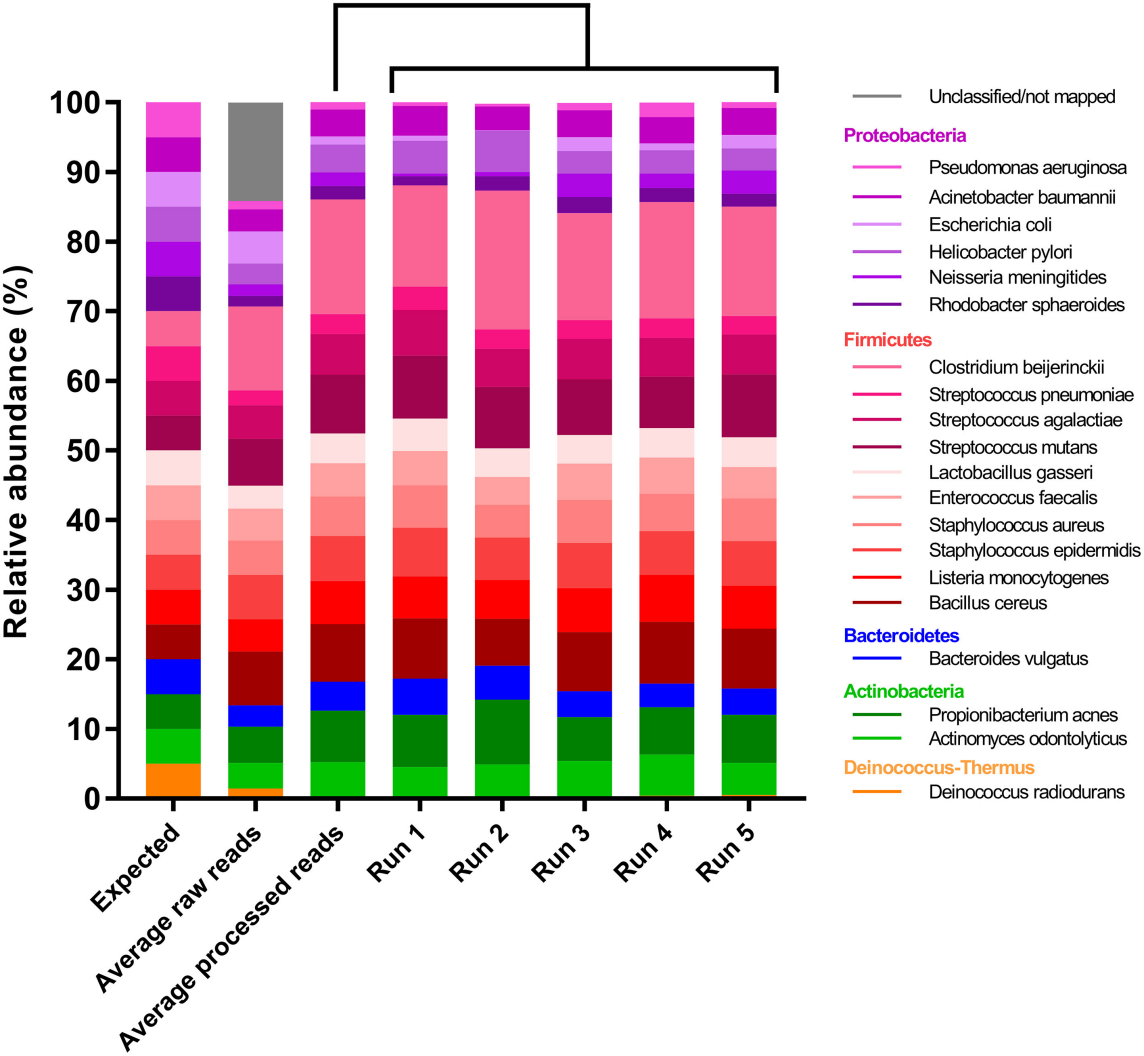
Relative abundance estimates of the 20-member mock community compared with the expected. Columns from left to right: Expected relative abundances, average relative abundances using the raw reads, average relative abundances using the processed reads and relative abundances in each of the five sequencing runs.

**Figure 2:**
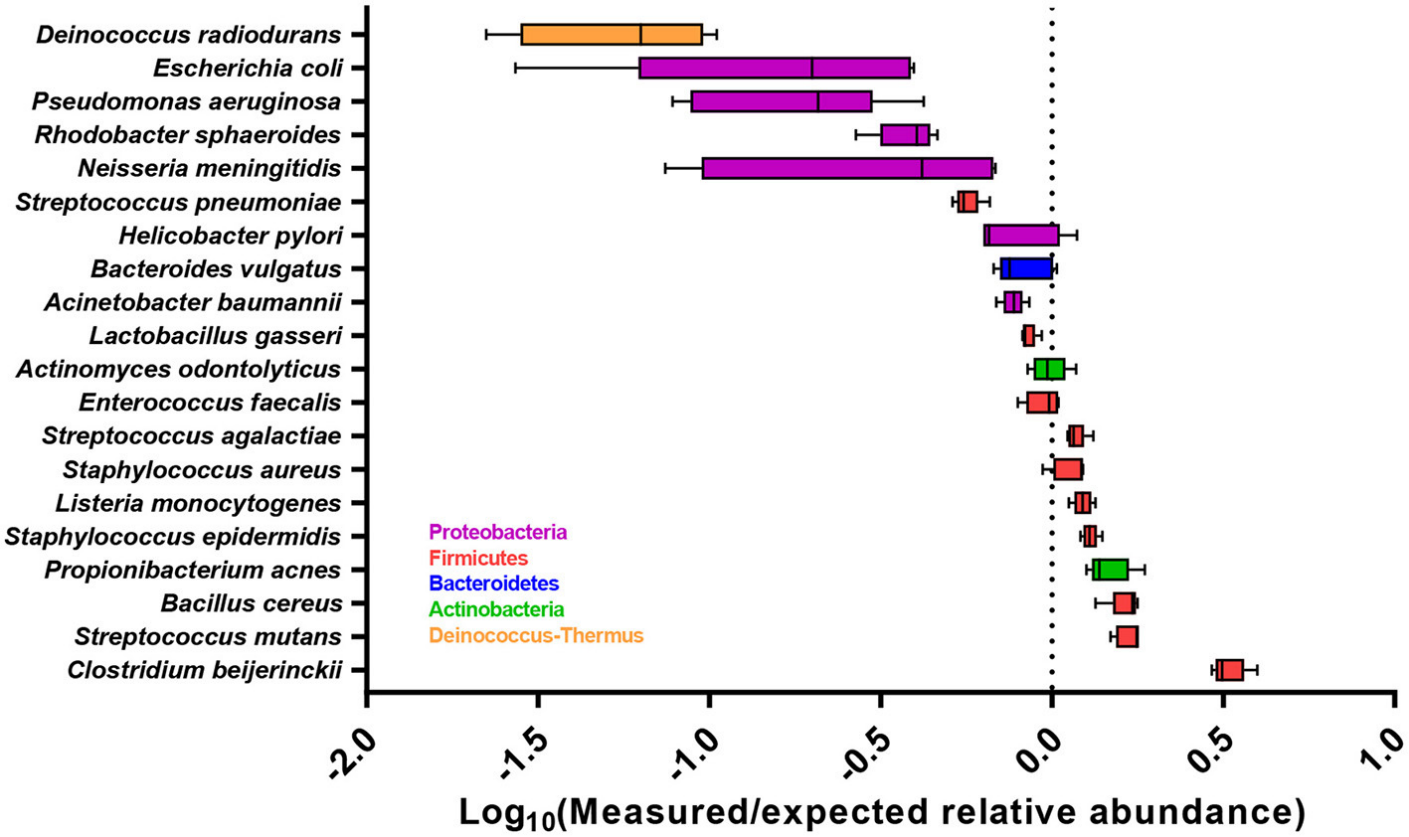
Accuracy of the abundance estimates across all five sequencing runs for each bacterial species, expressed as the Log_10_ ratio of measured relative abundance to the expected relative abundance. Boxplot show the median with 25 and 75 percentiles within the box and whiskers show the range. Dashed line indicates the expected relative abundance of 5%.

**Figure 3:**
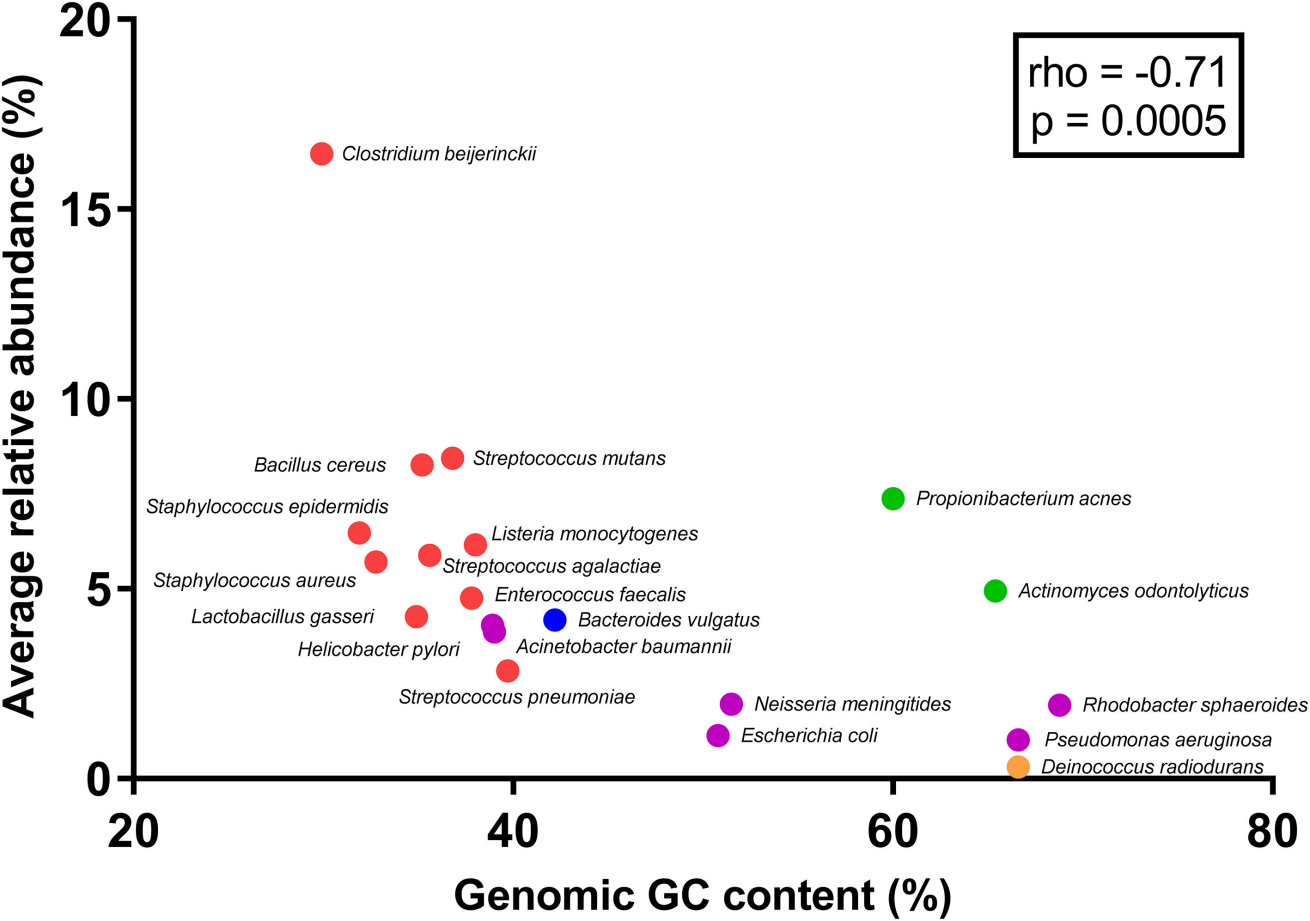
Correlation between genomic GC content and average abundance estimates for the 20-member mock community. Dots are colored according to phylum (Blue: Bacteroidetes, Purple: Proteobacteria, Green: Actinobacteria, Red: Firmicutes, and Yellow: Deinococcus-Thermus). Spearman's rank correlation coefficient (rho) and resulting *p*-value for the association is shown in the box.

**Figure 4:**
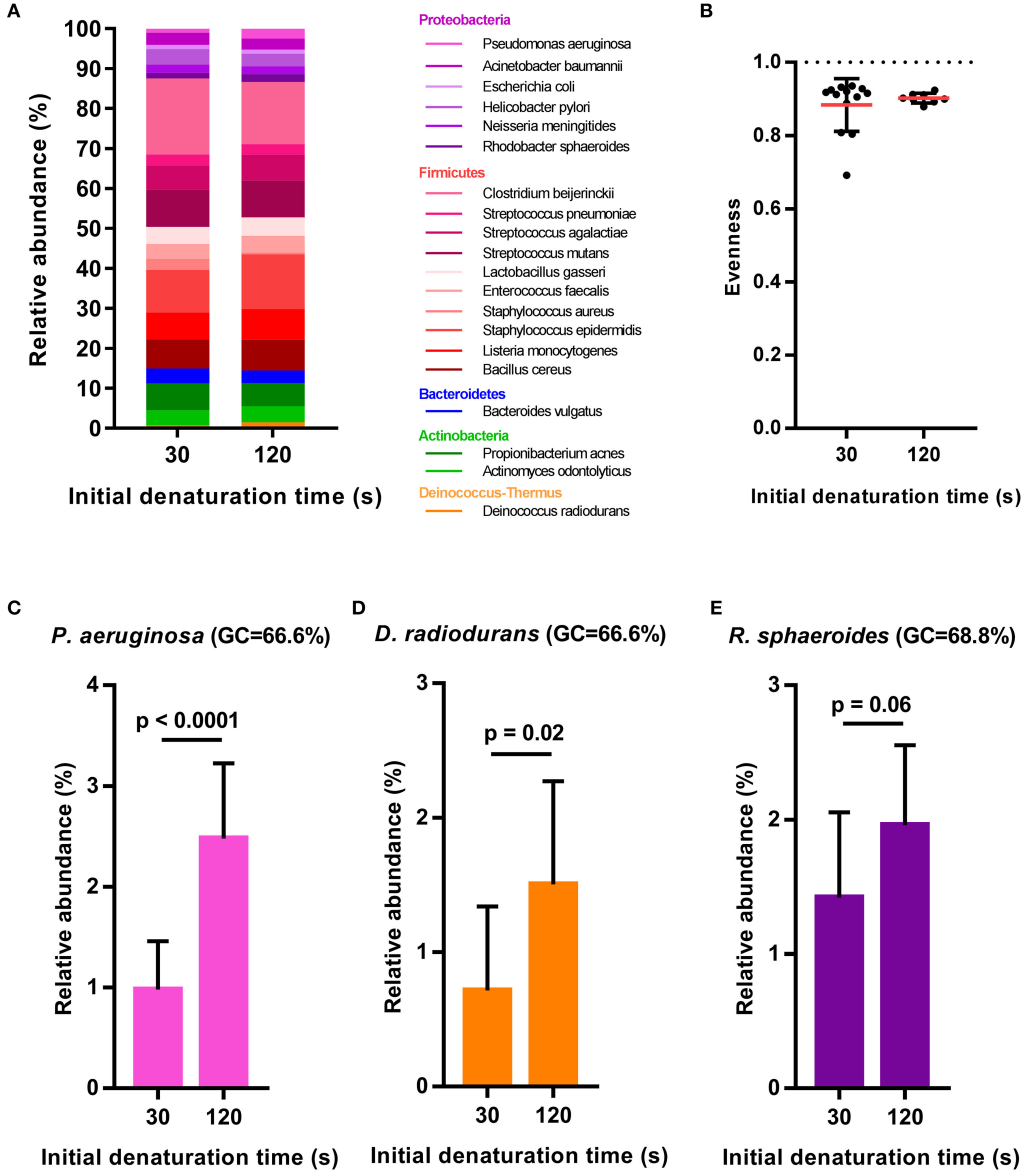
Relative abundance estimates of the 20-member mock community following different initial denaturing times of 30 s (*n* = 13) or 120 s (*n* = 8) during the library preparation PCR. **(A)** Bar plots of mean relative abundances of all mock community members, **(B)** Evenness of the mock community with dashed line indicating the expected evenness of 1 and **(C–E)** Bar plots showing mean relative abundance + sd for the three mock community members with highest GC-content. Statistical significance is evaluated by *t*-test.

After primer and length trimming as well as quality filtering, on average, 62.8% (range 53.4–70.5%) of the sequencing reads were retained (Table 1). Using the UPARSE pipeline (Edgar, 2013), a total of 31 non-chimeric OTUs were generated following de-novo clustering (Table 2). Collapsing OTUs into species level taxa based on BLAST against the 16S rRNA database, all of the 20 bacterial species were detected in all five separate sequencing runs (Figure 1, Tables 2, 3). The median coefficient of variation (CoV) was 11.8% (range 5.5–73.7%), with the majority of the species (15/20) having a CoV below 20% (Table 3). Generally, the determined relative abundances of Proteobacteria and *Deinococcus radiodurans* were underestimated, whereas those of species within Firmicutes (especially *C. beijerinckii*) were mostly overestimated compared with the expected community composition of 5% for each species (Figure 2, Table 3). It has previously been reported that premature read truncation associated with the semiconductor technology of Ion Torrent PGM may bias the community composition (Salipante et al., 2014). We also observed premature read truncation, which was the main reason for discarding approximately 30% (range 24.9–41.9%) of the raw reads during primer trimming. Since this may significantly contribute to community composition bias, we investigated the effect of relaxing our primer trimming criteria and abolished all length trimming and subsequent quality filtering (average 99.5% of the raw reads retained) and conducted the OTU analysis in UPARSE with 92.7% of the raw reads mapping to the newly generated OTUs. We then taxonomically classified the resulting 5328 OTUs, compiled the OTU abundance data to species level information and then compared the raw data to the processed (trimmed and quality filtered) data. We observed similar relative abundance estimates for the trimmed versus raw read comparison, with notable exceptions for *E. coli* (*p* = 0.0001, *t*-test) and *D. radiodurans* (*p* = 0.0003, *t*-test), which were significantly under-represented in the processed reads compared with the raw reads (Figure 1). We also found that the genomic GC% content of the 20 species in the mock community correlated negatively with the average relative abundance estimates following sequencing (Figure 3). In contrast, neither the GC-content of the full-length16S rRNA gene (rho = −0.29, *p* = 0.21) nor the specific amplified V3 region of the 16S rRNA gene (rho = 0.034, *p* = 0.89) correlated significantly with the average relative abundance estimates. This suggests that the genomic GC-content is an important contributor to bias when estimating relative abundance. PCR amplification bias in community composition may be caused by differences in genomic GC% of the community members since the double-stranded DNA of GC-rich organisms is more resistant to denaturation during PCR amplification (Polz and Cavanaugh, 1998). To explore this effect further, we conducted additional sequencing of the mock community after PCR amplification with increased initial denaturing time of 120 s (*n* = 8) compared with 30 s in the original PCR program (*n* = 13). Although results overall appeared similar (Figure 4A) with no difference in the overall evenness (Figure 4B), and the strength of the correlation between genomic GC-content and average relative abundance was only slightly reduced (rho = −0.60, *p* = 0.006), we did find that the average relative abundances of the three species with highest GC content was increased compared with the short denaturation time, reaching levels closer to the expected 5% (Figures 4C–E).

**Table 1:** Counts of raw sequencing reads, after primer/length trim and after quality filtering.

	**Raw reads (counts)**	**After trim (counts)**	**After trim (% retained)**	**After quality filtering (counts)**	**After quality filtering (% retained)**
Run 1	23,908	17,295	72.3	15,849	66.3
Run 2	19,258	13,673	71.0	12,525	65.0
Run 3	31,729	20,279	63.9	18,680	58.9
Run 4	53,484	40,148	75.1	37,721	70.5
Run 5	23,521	13,672	58.1	12,565	53.4
Average	30,380	21,013	69.2	19,468	62.8

**Table 2:** Collapsing of the 31 identified OTUs into the respective mock community species based on BLAST identity score against the 16S rRNA gene database at NCBI.

**No**.	**Species**	**OTU ID**	**BLAST Identity (%)**
1	*Acinetobacter baumannii*	OTU_12	100
2	*Actinomyces odontolyticus*	OTU_8	99
3	*Bacillus cereus*	OTU_2	100
4	*Bacteroides vulgatus*	OTU_14	100
		OTU_24	99
		OTU_31	97
5	*Clostridium beijerinckii*	OTU_1	100
		OTU_27	99
6	*Deinococcus radiodurans*	OTU_19	98
		OTU_29	99
7	*Enterococcus faecalis*	OTU_10	100
		OTU_28	98
8	*Escherichia coli*	OTU_18	100
		OTU_23	99
9	*Helicobacter pylori*	OTU_11	100
		OTU_26	98
10	*Lactobacillus gasseri*	OTU_9	100
11	*Listeria monocytogenes*	OTU_5	100
12	*Neisseria meningitidis*	OTU_16	100
		OTU_30	98
13	*Propionibacterium acnes*	OTU_3	100
14	*Pseudomonas aeruginosa*	OTU_17	100
		OTU_25	99
15	*Rhodobacter sphaeroides*	OTU_15	100
16	*Staphylococcus aureus*	OTU_6^a^	100
17	*Staphylococcus epidermidis*	OTU_20^b^	99
		OTU_21^b^	99
		OTU_22^b^	99
18	*Streptococcus agalactiae*	OTU_7^c^	100
19	*Streptococcus mutans*	OTU_4^d^	100
20	*Streptococcus pneumoniae*	OTU_13^e^	100

a *BLAST identity score against S. epidermidis = 98%*.

b *BLAST identity score against S. aureus = 97%*.

c *BLAST identity score against S. mutans < 97% and S. pneumoniae < 97%*.

d *BLAST identity score against S. agalactiae < 97% and S. pneumoniae < 97%*.

e *BLAST identity score against S. mutans < 97% and S. agalactiae < 97%*.

**Table 3:** Relative abundance estimates of the 20 species in the mock community in 5 separate runs and on average with coefficient of variation.

**Species**	**Relative abundance (%)**							**CoV (%)**
	**Expected**	**Run 1**	**Run 2**	**Run 3**	**Run 4**	**Run 5**	**Average**	
*Deinococcus radiodurans*	5.00	0.18	0.11	0.32	0.44	0.53	0.32	55.3
*Actinomyces odontolyticus*	5.00	4.25	4.85	5.11	5.88	4.60	4.94	12.5
*Propionibacterium acnes*	5.00	7.53	9.33	6.30	6.80	6.88	7.37	16.0
*Bacteroides vulgatus*	5.00	5.17	4.89	3.69	3.39	3.76	4.18	19.0
*Bacillus cereus*	5.00	8.68	6.70	8.47	8.89	8.59	8.27	10.8
*Listeria monocytogenes*	5.00	6.03	5.60	6.32	6.69	6.15	6.16	6.5
*Staphylococcus epidermidis*	5.00	7.02	6.05	6.53	6.30	6.43	6.47	5.5
*Staphylococcus aureus*	5.00	6.13	4.70	6.16	5.44	6.11	5.71	11.2
*Enterococcus faecalis*	5.00	4.90	3.98	5.23	5.21	4.46	4.76	11.2
*Lactobacillus gasseri*	5.00	4.67	4.10	4.12	4.17	4.27	4.27	5.5
*Streptococcus mutans*	5.00	8.98	8.83	8.01	7.41	8.97	8.44	8.3
*Streptococcus agalactiae*	5.00	6.60	5.55	5.82	5.64	5.78	5.88	7.1
*Streptococcus pneumoniae*	5.00	3.29	2.83	2.73	2.76	2.56	2.83	9.7
*Clostridium beijerinckii*	5.00	14.64	19.88	15.41	16.68	15.68	16.46	12.4
*Rhodobacter sphaeroides*	5.00	1.34	2.10	2.32	2.02	1.86	1.93	19.1
*Neisseria meningitides*	5.00	0.37	0.61	3.42	2.09	3.33	1.96	73.7
*Helicobacter pylori*	5.00	4.71	5.92	3.15	3.28	3.15	4.04	30.7
*Escherichia coli*	5.00	0.71	0.14	1.98	1.00	1.90	1.15	68.8
*Acinetobacter baumannii*	5.00	4.30	3.44	3.87	3.80	3.91	3.86	7.9
*Pseudomonas aeruginosa*	5.00	0.50	0.39	1.04	2.11	1.07	1.02	66.7

## Discussion

We used a simple strategy for library preparation based on PCR amplification of the V3-region of the 16S rRNA gene and subsequent amplicon sequencing with the Ion Torrent PGM platform. This strategy is limited by the fact that species level classification may not always possible in more complex natural bacterial populations. ThermoFisher offers a standard “Ion 16S Metagenomics Kit” for library preparation prior to sequencing on the Ion Torrent PGM, which is based on sets of primers targeting several hypervariable regions of the 16S rRNA gene (V2, V3, V4, V6, V7, V8, and V9). While that strategy has the benefit of higher resolution, is also more laborious and expensive and the data is not currently possible to analyse with commonly used pipelines such as those implemented in UPARSE (Edgar, 2013), QIIME (Caporaso et al., 2010), or mothur (Schloss et al., 2009), which limits its usefulness in microbial ecology studies. The use of validated bacterial mock communities from BEI Resources to investigate the overall performance of various 16S rRNA gene sequencing strategies has previously been reported (Salipante et al., 2014; Fouhy et al., 2016). These studies have focused on DNA extraction procedures, choice of PCR primers, selection of target variable regions within the 16S rRNA gene and choice of sequencing platform (MiSeq versus IonTorrent PGM) as the major variables that may affect the outcome. Salipante et al. sequenced the V1-2 region of the 16S rRNA gene in the equimolar 20-species mock community and found a higher error rate (especially in homopolymeric regions) from the Ion Torrent PGM sequencing data set compared with the corresponding Illumina MiSeq sequencing data set (Salipante et al., 2014). It is important to note that since the publication by Salipante et al., the Ion Torrent Hi-Q chemistry has been introduced, which significantly improves the sequence read quality and error rates in homopolymeric regions (Churchill et al., 2016; Pereira et al., 2016). In the study by Salipante et al., the authors also noted the deviation in relative abundance from the expected 5% for some of the included species. This was partly attributed to premature read truncation associated with the semiconductor technology employed by Ion Torrent PGM (Salipante et al., 2014). This premature read truncation occurred preferentially with specific species of the mock community such as *P. acnes* and *A. odontolyticus*, but was apparently very dependent on the sequencing direction, as only reverse direction sequencing resulted in notable underestimation of the mentioned strains within the community (Salipante et al., 2014). In the present study, we also observed premature read truncation on the average of ~30% of the raw reads. Although almost half of these truncated reads (14.2% of the total raw reads) could not sufficiently be classified or failed to map to the generated OTUs, the inclusion of the remaining half affected the relative abundance estimates of a few species when comparing the trimmed and processed data. Specifically, *E. coli* and *D. radiodurans* were better represented among the raw reads compared with the processed reads, suggesting bias toward premature truncation of these sequences. It is important to note that such biases would probably depend on the selected target sequence (e.g., the variable region of 16S rRNA that is used), which may explain the differences observed between the two studies.

Fouhy et al. showed that choice of primers (degenerate vs. non-degenerate) affect the inferred community composition following sequencing of the same mock community (Fouhy et al., 2016). Indeed, a higher binding efficiency of GC-rich permutations of degenerate primers can contribute to a skew in community composition (Wagner et al., 1994), which is avoided by using non-degenerate primers. However, even a few mismatches between primers and a target sequence in a template pool from different organisms can contribute to an underestimation of the relative representation of that specific sequence (Hongoh et al., 2003). Thus, for accurate abundance estimation, the use of non-degenerate primers and assurance of near perfect primer match to all expected targets of the microbial community profiled, is desirable. In the present study we use non-degenerative primers, which have been validated against members of the five predominant bacterial phyla present in the gastro-intestinal tract. These primers are almost identical (maximum 1 mismatch) to the corresponding region in more than 98% of the type species belonging to Firmicutes, Bacteroidetes, Proteobacteria, and Actinobacteria; however, there is a slightly lower identity to members of the Verrucomicrobia including *Akkermansia muciniphila*. Local GC-content of the target gene (16S rRNA gene) or the specific amplified region of the target gene has previously been shown to contribute to PCR bias (Polz and Cavanaugh, 1998). However, we found no significant correlations between full length or V3 region 16S rRNA gene GC-content and relative abundance estimates of the mock community species. This may be due to the fact that the V3 region amplicon is relatively short (180-200 bp) and the low number of PCR cycles (24) implemented in our protocol. PCR bias may also result from preferential denaturation of sequences within low overall genome GC content (Polz and Cavanaugh, 1998), which likely explains the negative correlation between whole genome GC-content and relative abundance estimates of the mock community members. Indeed, when we increased the initial denaturing time from 30 s (in the original PCR program) to 120 s, the relative abundance estimates of the three mock community species with the highest genomic GC-content was clearly increased although the overall evenness did not change and the correlation between genomic GC-content and relative abundance estimates was only slightly reduced. Thus, simply increasing the initial denaturing time is not sufficient to completely circumvent this issue and further optimization of the PCR procedure, e.g., addition of DMSO to aid denaturation or removal of Mg^2+^ to reduce double-stranded DNA stability, may be useful to further correct these discrepancies. Although the reproducibility was generally good, we observed that it varied considerably between mock-community members. Whereas the majority (15 out of the 20 mock community species) had a CoV below 20%, the underestimated species (Proteobacteria and *Deinococcus radiodurans*) showed higher CoV. Since these are also among the species with high GC-content (Figure 3) it may be connected to variable denaturation of their genomic DNA across PCR runs.

## Conclusion

Ion Torrent PGM 16S rRNA gene sequencing of a 20-species mock community appeared reproducible and had a median coefficient of variation of 11.8% in relative abundance across five separate sequencing runs. The observed inaccuracies in abundance estimates compared with the expected are partly explained by premature read truncation, but more pronounced by PCR bias, caused by differences in genomic GC content. Therefore, optimizing PCR conditions during library preparation is important to obtain accurate results.

## Author contributions

ML and MB designed the study. ML performed 16S rRNA amplicon library preparation. MD performed the Ion Torrent PGM sequencing. ML and MB analyzed and interpreted the data. ML drafted the manuscript and all authors read and approved the final version.

### Conflict of interest statement

The authors declare that the research was conducted in the absence of any commercial or financial relationships that could be construed as a potential conflict of interest. The reviewer AGN and handling Editor declared their shared affiliation.
