# Corrigendum: Genomic GC-Content Affects the Accuracy of 16S rRNA Gene Sequencing Based Microbial Profiling due to PCR Bias

**DOI:** 10.3389/fmicb.2022.878696

**Published:** 2022-03-18

**Authors:** Martin F. Laursen, Marlene D. Dalgaard, Martin I. Bahl

**Affiliations:** ^1^Division of Diet, Disease Prevention and Toxicology, National Food Institute, Technical University of Denmark, Kongens Lyngby, Denmark; ^2^Department of Biotechnology and Biomedicine, Technical University of Denmark, Kongens Lyngby, Denmark

**Keywords:** ion torrent PGM, 16S rRNA gene sequencing, reproducibility, accuracy, mock community, genomic GC content

In the original article, there was a mistake in the legend and image for Figure 2 as published. The legend incorrectly states Log2 ratio and the x-axis label in the image incorrectly states “Log2(Measured/expected relative abundance)”. The corrected legend and image for Figure 2 appears below.

**Figure 2:**
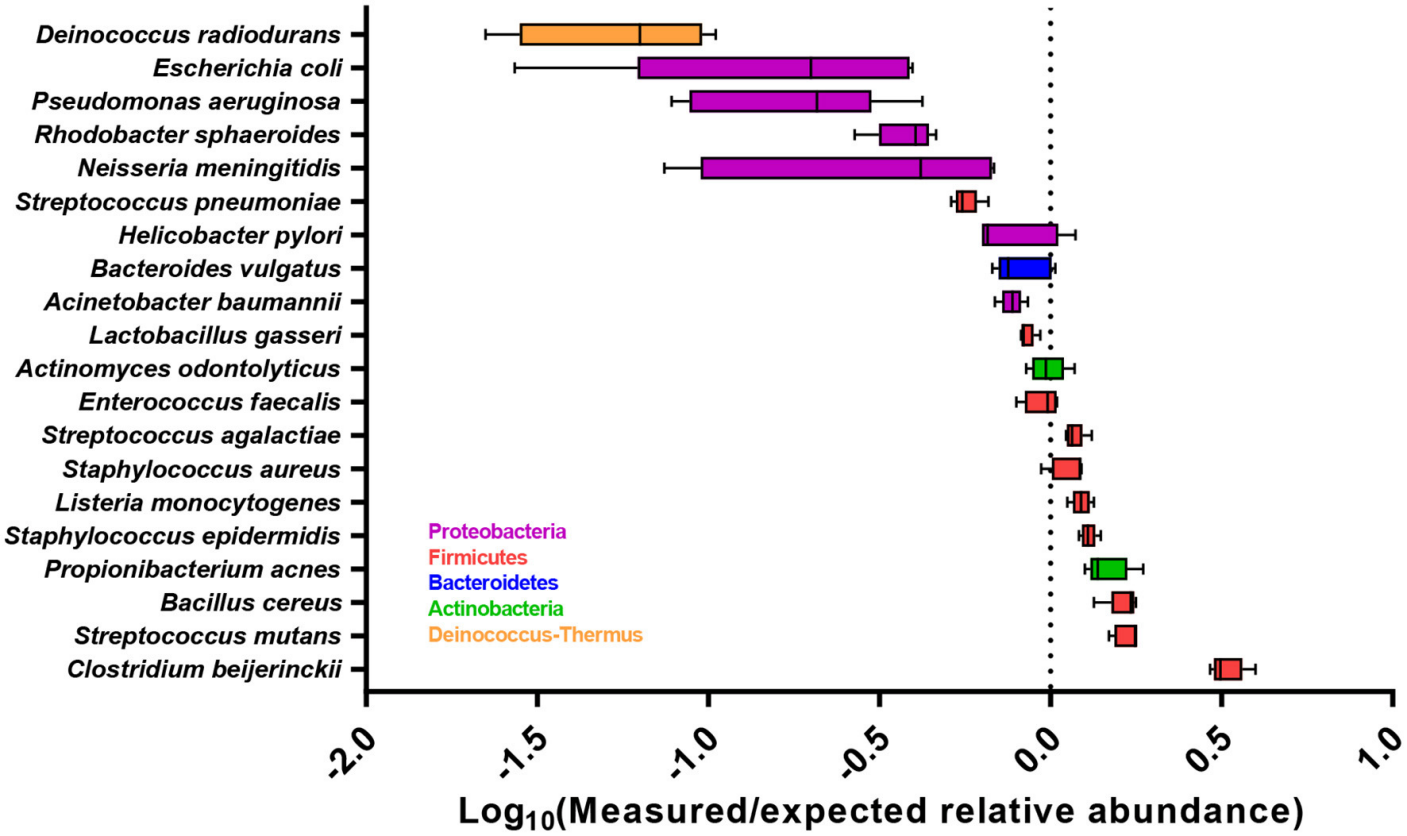
Accuracy of the abundance estimates across all five sequencing runs for each bacterial species, expressed as the Log_10_ ratio of measured relative abundance to the expected relative abundance. Boxplot show the median with 25 and 75 percentiles within the box and whiskers show the range. Dashed line indicates the expected relative abundance of 5%.

The authors apologize for this error and state that this does not change the scientific conclusions of the article in any way. The original article has been updated.

## Publisher's Note

All claims expressed in this article are solely those of the authors and do not necessarily represent those of their affiliated organizations, or those of the publisher, the editors and the reviewers. Any product that may be evaluated in this article, or claim that may be made by its manufacturer, is not guaranteed or endorsed by the publisher.

